# Transcriptome Sequencing of the Diatom *Asterionellopsis thurstonii* and In Silico Identification of Enzymes Potentially Involved in the Synthesis of Bioactive Molecules

**DOI:** 10.3390/md21020126

**Published:** 2023-02-15

**Authors:** Eleonora Montuori, Kevin A. Martinez, Daniele De Luca, Adrianna Ianora, Chiara Lauritano

**Affiliations:** 1 Department of Chemical, Biological, Pharmaceutical and Environmental Sciences, University of Messina, Viale F. Stagno d’Alcontres 31, 98166 Messina, Italy; 2 Ecosustainable Marine Biotechnology Department, Stazione Zoologica Anton Dohrn, Via Acton 55, 80133 Naples, Italy; 3 Department of Biology, University of Naples Federico II, Via Foria 223, 80139 Naples, Italy

**Keywords:** *Asterionellopsis thurstonii*, biosynthetic pathways, enzymes, transcriptome sequencing, transcriptome mining

## Abstract

Microalgae produce a plethora of primary and secondary metabolites with possible applications in several market sectors, including cosmetics, human nutrition, aquaculture, biodiesel production and treatment/prevention of human diseases. Diatoms, in particular, are the most diversified microalgal group, many species of which are known to have anti-cancer, anti-oxidant, anti-diabetes, anti-inflammatory and immunomodulatory properties. Compounds responsible for these activities are often still unknown. The aim of this study was to de novo sequence the full transcriptome of two strains of the diatom *Asterionellopsis thurstonii*, sampled from two different locations and cultured in both control and phosphate starvation conditions. We used an RNA-sequencing approach to in silico identify transcripts potentially involved in the synthesis/degradation of compounds with anti-cancer and immunomodulatory properties. We identified transcript coding for L-asparaginase I, polyketide cyclase/dehydrase, bifunctional polyketide phosphatase/kinase, 1-deoxy-D-xylulose-5-phosphate synthase (fragment), inositol polyphosphate 5-phosphatase INPP5B/F, catechol O-Methyltransferase, digalactosyldiacylglycerol synthase (DGD1), 1,2-diacylglycerol-3-beta-galactosyltransferase and glycerolphosphodiester phosphodiesterase. Differential expression analysis also allowed to identify in which culturing condition these enzymes are more expressed. Overall, these data give new insights on the annotation of diatom genes, enzymatic pathways involved in the generation of bioactive molecules and possible exploitation of *Asterionellopsis thurstonii*.

## 1. Introduction

Microalgae produce a plethora of primary and secondary metabolites and in vitro and in vivo screenings have shown that some of these compounds have interesting bioactivities for the prevention and/or treatment of human diseases [1]. Diatoms, in particular, are the most various and abundant group of microalgae in terms of species [2]. Many species have been shown to have anti-cancer [3], anti-oxidant [4], anti-diabetes [5], anti-inflammatory [4] and immunomodulatory properties [6], but the compounds responsible for these activities are often still unknown. Recently, several studies have focused on the study of enzymatic pathways involved in the biosynthesis of these compounds to understand how these compounds are synthetized and under which culturing/environmental conditions [7,8,9], coupled with -omics techniques (e.g., genomics and transcriptomics), and heterologous expression or genetic engineering to increase the production of compounds with biological activity [1]. To date, the number of microalgal genomes sequenced are still few compared with the huge number of existing microalgae [10]. Available diatom genomes are those of *Conticribra weissflogii*, *Fistulifera solaris*, *Fragilariopsis cylindrus*, *Nitzschia putrida*, *Phaeodactylum tricornutum*, *Pseudo-nitzschia multiseries*, *Pseudo-nitzschia multistriata*, *Seminavis robusta*, *Skeletonema costatum*, *Thalassiosira oceanica* and *Thalassiosira pseudonana* [10,11,12,13]. In addition, there is a current project that will sequence 100 diatom species (https://isdr.org/embarking-on-the-100-diatom-genomes-project/; accessed on 6 October 2022).

Several studies have shown that, depending on the culturing conditions, diatoms may activate specific metabolic pathways and produce key secondary metabolites with important ecological roles, as well as potential applications for human health. For instance, it has been shown that, in nutrient starvation, the diatom *Skeletonema marinoi* produces higher amounts of secondary metabolites called polyunsaturated aldehydes (PUAs) [14], which have deterrent activity for predators as well as anti-cancer properties [15]. The experimental approach consisting of using different culturing conditions known as OSMAC (“One Strain Many Compounds”; [16]) has been used to improve the production of compounds of interest. For example, Lauritano et al. [17] showed that specific microalgal clones were active for anti-inflammatory applications only when cultivated in control conditions (repleted medium, i.e., for the diatoms *Cylindrotheca closterium*, *Pseudo-nitzschia pseudodelicatissima* and *Trieres mobiliensis*) while others were active only in stressful conditions (i.e., nutrient starvation), such as the diatom *Skeletonema marinoi* (active against human melanoma cells or pathogenic bacteria).

**Figure 1:**
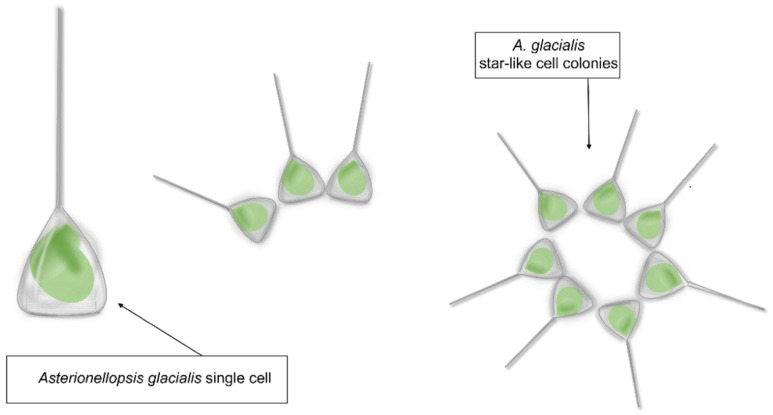
*Asterionellopsis glacialis* sensu lato shape representation.

*Asterionellopsis glacialis* is a diatom species belonging to the family Asterionellopsidaceae. *Asterionellopsis* spp. cells are three-cornered in shape with an enlarged narrow region and very delicate transapical striae (Figure 1) [18].

*Asterionellopsis glacialis* has been considered for a long time as a cosmopolitan and eurytopic (i.e., able to tolerate a wide range of environmental conditions) species, often abundant in phytoplankton communities of cold to temperate waters [19,20]. A recent taxonomic study by Kaczmarska et al. [21], aiming at testing the conspecificity of geographically distant strains of *A. glacialis*, has revealed the occurrence of extensive cryptic diversity and led to the description of four new species. However, these new findings have not been incorporated into already existing or new -omic data deposited in public repositories, with relevant implications for biotechnological studies. For instance, within the Marine Microbial Eukaryte Transcriptome Sequencing Project [22] (MMETSP; https://www.imicrobe.us/; accessed on 14 October 2022), there are three transcriptomes deposited as *A. glacialis* that, according to Kaczmarska et al. [21], may be attributed to *A.* cf. *glacialis* (CCMP134), *A. thurstonii* (CCMP1581) and probably to *A. maritima* (strain from Martha’s Vineyard Coastal Observatory site) according to the sampling locality. Similarly, the whole genome deposited in GenBank as *Asterionellopsis glacialis* strain A3 (collected in July 2016 along the coast of the Arabian Gulf as reported on https://www.ncbi.nlm.nih.gov/nuccore/1930234978; accessed on 6 October 2022) needs to be confirmed as such in the framework of these new taxonomic findings. The taxonomic characterization of the strains under study is a fundamental prerequisite. The advantage of using microalgae in marine drug discovery is associated with their metabolic plasticity, associated in turn with the production of compounds that can find applications in different biotechnology sectors [17,23] and with the possibility of cultivating them in large quantities in an eco-friendly and eco-sustainable way.

The biological activity of *Asterionellopsis* has been poorly explored. In 2005, Wichard and collaborators showed the presence of PUAs in *Asterionellopsis glacialis* [24], known to reduce reproductive success of copepods and other invertebrates and to induce allelopathic effects on other phytoplankton species and variations in the community composition of picoplankton, with possible consequences on the marine food web structure [25,26,27,28]. Rörig et al. [29] found difficulties in *Asterionellopsis* cultivation, showing that the strains, after weeks or months of laboratory culturing, started losing their viability and presented aberrant frustules. They suggested a reduction in nutrients or a low quantity of salts in the culture medium as possible causes for these effects [29]. Saturated and polyunsaturated fatty acids (PUFA) have also been found in some strains of *Asterionellopsis glacialis* sensu lato (s.l.), as shown in the work of Viso et al. [30] and Rörig et al. [29]. Various *Asterionellopsis* strains may also have a different content of lipids and fatty acids related to genetic and chemical diversity of strains [21,29]. In addition, Shibl et al. [31] also showed that *A. glacialis* reprogrammed its transcriptional and metabolic profile in the presence of bacteria, secreting a series of primary metabolites and two unusual metabolites, rosmarinic acid and azelaic acid [31]. Lauritano et al. [32] also tested *A. glacialis* for possible anti-bacterial activity but did not find positive hits. To our knowledge, a transcriptomic approach has never been used for *A. glacialis* s.l. in order to identify bioactive biosynthetic pathways for the possible biotechnological exploitation of this cosmopolitan diatom [33].

In this work, we have sequenced, de novo assembled and analyzed the transcriptome of two strains of *A. glacialis* s.l.: strain A4, collected from the English Channel (waters off Dunkirk, France), and strain FE355, collected from the Gulf of Naples (Italy). In the current study, we first characterized the strains with a DNA barcoding approach. Then, we sequenced the transcriptome of these strains both in control and phosphate starvation conditions. Phosphate starvation was selected as the stress condition because it is known to influence the production of active/toxic compounds [8,34,35]. For example, phosphorus deficiency was found to induce a significant increase in total fatty acids in the marine diatom *Phaeodactylum tricornutum* and the green alga *Dunaliella tertiolecta*. Similarly, Frangópulos and collaborators showed an increased production of toxins by the dinoflagellate *Alexandrium* spp. under phosphorus limitation [34]. In parallel, the stationary phase was selected in the current study for arresting algal growth because previous studies have shown that this phase is when algae start producing higher amounts of secondary metabolites with possible bioactivities [14]. The final goal was to identify enzymes that can be involved in the synthesis/degradation of compounds with immunomodulatory and anti-cancer properties.

## 2. Results

### 2.1. Genetic Characterization of the Strains

A Bayesian phylogenetic tree based on the analysis of the ITS region showed that the strains used in the present study belong to *A. thurstonii* (posterior probability, p.p. = 1; Supplementary Material Figure S1) and that they are identical to the strains collected in the Black Sea and at Renesee (about 120 km from where our strain A4 was collected). The tree was inferred under the GTR + G model using a final alignment of 658 bp (Data S1).

### 2.2. Transcriptome Assembly and Funtional Annotation

RNA-sequencing was performed for six samples for each strain and three for each culturing condition (i.e., phosphate starvation and control). Prior to further analysis, a quality check was performed on the raw sequencing data, removing low-quality portions while preserving the longest high-quality part of the next generation sequencing reads. The minimum length was set to 35 bp and the quality score to 25. The software BBDuk (BBTools User Guide DOE Joint Genome Institute. Available online: https://jgi.doe.gov/data-and-tools/software-tools/bbtools/bb-tools-user-guide/; accessed on 10 April 2020) was used for this scope. Quality of the reads was checked before and after the trimming step. The assembly was performed with Trinity (v2.8.6) [36] using the trimmed pair reads as input. The number of obtained transcripts was 44,430 for FE355 and 46,835 for A4. At this point, the following steps were performed in order to refine and evaluate the assembly: (1) redundancy removal with CD-HIT-EST [37]; (2) remove zero count transcripts with Kallisto [38]; (3) remove contamination with BLAST (National Library of Medicine, Rockville Pike Bethesda, MD, USA); (4) evaluate with Transrate [39] (Table 1).

The final number of transcripts obtained was 40,493 for FE355 and 41,698 for A4. The transcriptome statistics are reported in Table 2 for FE355 and A4, respectively.

The first annotation step included the identification of open reading frames (ORFs) across the transcript sequences. The second step involved the conversion of transcript sequences to protein sequences. The third step included the annotation of the functional description (DE; Supplementary Material Table S1 for FE355 and Table S2 for A4) and GO classes (by using TransDecoder, available online at https://github.com/TransDecoder/TransDecoder/releases, accessed on 10 April 2020, and PANNZER tools [40]). A total number of 23,663 proteins was found for FE355 and 25,098 for A4, respectively.

### 2.3. Differential Expression Analysis

A differential analysis of transcript expression was performed with the following comparison: (i) A4_phosphate starvation vs. A4_Control, (ii) FE355_ phosphate starvation vs. FE355_Control and (iii) A4_Control vs. FE355_Control (Supplementary Material Tables S3–S5, respectively). The software Kallisto (v0.46.0) [38] was used for transcript expression quantification and the NOISeq [41,42] package to perform differential analysis with RNA-seq data. The following table shows the number of differentially expressed transcripts (up-regulated and down-regulated) in each comparison. Table 3 shows only transcripts that were significantly differentially expressed, i.e., with an FDR ≤ 0.001 and log_2_FC ≥ 1 and log_2_FC ≤ −1.

Finally, for the significantly differentially expressed transcripts, a gene ontology enrichment analysis (GOEA) was performed to identify the most enriched gene ontology (GO) categories that were up-regulated (Supplementary Material Table S6) and down-regulated (Supplementary Material Table S7). In particular, fatty acid biosynthetic processes and signal transductions were the most enriched up-regulated biological processes (BP), together with bounding membrane of organelle as cellular component (CC) and lipid binding as molecular function (MF) in A4 in control condition versus FE355 in control condition. For A4 in phosphate starvation versus its control, glucose metabolic process (BP), host cell nucleus (CC) and glucose-6-phosphate dehydrogenase activity (MF) were the most enriched up-regulated GO, whereas for FE355 in phosphate starvation versus its control there were cysteine transport (BP), glycosome (CC) and phosphatidylserine binding (MF) (Supplementary Material Table S6). Regarding the most enriched down-regulated GO, there were toll signaling pathway (BP), synapse (CC) and proteasome binding (MF) in A4 in control condition versus FE355 in control condition. For A4 in phosphate starvation versus its control, the most enriched down-regulated GO were response to high light intensity (BP), photosystem II oxygen evolving complex (CC) and protein transmembrane transporter activity (MF), while cell morphogenesis (BP), phagocytic vesicle (CC), aminoacyl-tRNA editing activity (MF) were down-regulated for FE355 in phosphate starvation versus its control (Supplementary Material Table S7).

#### 2.3.1. Differential Expression Analysis of Strain A4

From the total number of 25,098 proteins found in A4, 2276 were up-regulated and 1393 were down-regulated; only 2385 of these were annotated. Table 4 lists the first ten up-regulated and down-regulated genes with NCBI NR assignment in the *A. thurstonii* A4 strain grown in phosphate starvation with respect to the control culturing condition.

The up-regulated genes in Table 4 included O-methyltransferase family 3, which is a part of an S-adenosyl-l-methionine-dependent O-methyltransferase family (enzymes that have been mainly studied in citrus fruits [43]). These enzymes bind S-adenosyl-l-methionine (SAM) to catalyze the O-methylation of various secondary metabolites that have a broad spectrum of biological activities, including anti-inflammatory, anti-carcinogenic and anti-atherogenic properties [43]. Other up-regulated genes were alkaline phosphatase ALP, a monomeric enzyme of 86 kDa that in algae is known to hydrolyze dissolved organophosphate (DOP) in order to obtain a phosphorus when the preferred dissolved inorganic phosphorus (DIP) is present in limited supply [44]; catechol O-methyltransferase domain-containing protein 1 involved in the catalysis of the transfer of a methyl group to the oxygen atom of an acceptor molecule [45]; glycerophosphodiester phosphodiesterase (GD-PD) involved in the degradation of various glycerophosphodiesters to glycerol-3 phosphate and corresponding alcohol moiety [46]; GD-PD, a key enzyme in the phospholipid metabolism pathway because it controls the concentration of glycerol-3 phosphate that is fundamental for phospholipid remodeling and synthesis [46,47]. Microalgal phospholipids are known to exert various bioactivities such as anti-viral, anti-microbial and anti-tumor activity in vitro [48]. The ten most up-regulated genes also included acyl-CoA thioesterase (ACOT) (which catalyzes the hydrolysis of acyl-CoA into free fatty acids and coenzyme A (CoASH) regulating their respective intracellular levels [49]), solute carrier family 35, member C2 (SLC35C2) (which is ubiquitously expressed in humans although the level of expression is tissue dependent (https://www.uniprot.org/uniprotkb/Q9NQQ7/entry; accessed on 2 October 2022)) and DNA degradation protein EddB, which is an enzyme with catalytic activity of endo/exonuclease/phosphatase and is involved in homophilic cell adhesion via plasma membrane adhesion molecules (https://www.uniprot.org/uniprotkb/A0A2D6VNM2/entry; accessed on 20 September 2022).

The ten most down-regulated genes included peptidyl-prolyl cis-trans isomerase and peptidyl-prolyl cis-trans isomerase, chloroplastic-like isoform X2, which are a superfamily of ubiquitous folding catalysts [50]. These enzymes catalyze the cis–trans isomerization of the N-terminal peptide bond to proline residues in polypeptide chains [51] and are highly conserved in bacteria, plants and mammals [52]. This transcript was down-regulated also in FE355 cultured in phosphate starvation. Phosphoglycerate mutase-like protein is an ubiquitous enzyme that catalyzes phase eight of glycolysis by transferring phosphorus from carbon 3 to carbon 5, giving 2-phosphoglycerate and 3-phosphoglycerate [53]. Pentapeptide repeat-containing proteins are a family of proteins of over 500 members in the prokaryotic and eukaryotic kingdoms, but the biochemical function of most of them is still unknown [54]. The thylakoid lumenal 17.4 kDa protein, chloroplastic, is a thylakoid lumen protein. Its family is characterized by the presence of many tandem repetitions of pentapeptides [55]. 3-beta hydroxysteroid dehydrogenase/isomerase family catalyzes the biosynthesis of steroid progesterone [56]. 3-oxoacyl-ACP synthase (fragment) is involved in fatty acid synthesis [57], known to be one of the main components of microalgae biomass, with possible nutraceutical applications [58]. Finally, DUF1995 domain-containing protein (fragment) is part of a series of proteins whose function is not known and for this reason they are named DUF (domains of unknown function) [59] and calcium/calmodulin dependent protein kinase II association-domain protein is an enzyme known to be involved in the assembly of single proteins into large (8 to 14 subunits) multimers [60].

#### 2.3.2. Differential Expression Analysis of Strain FE355

From the total number of 23,663 proteins found in FE355, 2206 were up-regulated and 1152 were down-regulated; only 2572 of these are annotated. Table 5 lists the ten most up- and down-regulated genes in the *A. thurstonii* FE355 strain grown in phosphate starvation conditions with respect to the control.

The ten most up-regulated genes included the transcriptional regulator MraZ which is a DNA-binding transcriptional regulator (https://www.uniprot.org/uniprotkb/O83398/entry#family_and_domains; accessed on 20 September 2022) [61]; ribosomal protein l12, which is an essential protein that forms an important functional domain in the ribosome involved in interactions with translation factors during protein biosynthesis [62]; translation initiation factor IF-3, which is a translation regulation factor essential for the development of organisms because its function is to increase the accuracy of the start codon selection [63]; the SPX domain-containing protein, known to be essential for maintaining inorganic phosphate homeostasis in plants [64]. The SPX domain-containing protein is also known to regulate the synthesis of anthocyanins, which are pigments known for their anti-oxidant and anti-cancer properties [64]. Finally, the ten most up-regulated genes included “cell division cycle 2, cofactor of the anaphase promoter complex (APC complex)” which is essential for cell division, particularly during anaphase, and has a positive role in the regulation of the ubiquitination process by interacting and activating the ubiquitin-protein transferase (https://www.uniprot.org/uniprotkb/A0A177EC18/entry; accessed on 20 September 2022).

Regarding the ten most down-regulated genes in *A. thurstonii* FE355, there were some light-harvesting complex proteins, such as light-harvesting complex protein LHCC4 (Fragment), light-harvesting complex I polypeptide, light harvesting complex protein 10, chloroplast light-harvesting protein isoform 7, chloroplast light-harvesting protein isoform 5 and fucoxanthin-chlorophyll a/c light-harvesting protein (fragment). In general, light-harvesting complex proteins are a superfamily of proteins that are involved in photosynthesis by binding carotenoids and chlorophyll that adsorbs light and transfers energy to one chlorophyll a molecule at the reaction center of the photosystem [65,66]. The transcript of fucoxanthin chlorophyll a/c protein was down-regulated in FE355.

Finally, the last three most down-regulated genes were: protochlorophyllide oxidoreductase, which is an enzyme that catalyzes the reduction of protochlorophyllide to chlorophyllide in the penultimate step of biosynthesis of chlorophyll required for photosynthetic light absorption and energy conversion [67]; pyruvate carboxylase (fragment), which is a member of the family of biotin dependent carboxylases that catalyzes the ATP-dependent carboxylation of pyruvate to form oxaloacetate which may be utilized in the synthesis of glucose, fat, some amino acids or their derivatives and several neurotransmitters [68]); iron-only hydrogenase group A, which catalyzes the reversible oxidation of molecular hydrogen (H2) [69].

### 2.4. DEGs Involved in Anti-Cancer and Immunomodulatory Responses

In both the annotation of A4 and FE355, we found the expression of the following transcripts that may be involved in the synthesis/degradation of compounds with anti-cancer and immunomodulatory responses: L-asparaginase I, polyketide cyclase/dehydrase, bifunctional polyketide phosphatase/kinase, 1-deoxy-D-xylulose-5-phosphate synthase (fragment), inositol polyphosphate 5-phosphatase INPP5B/F, catechol O-Methyltransferase, digalactosyldiacylglycerol synthase (DGD1), 1,2-diacylglycerol-3-beta-galactosyltransferase and glycerolphosphodiester phosphodiesterase.

L-asparaginase I belongs to the family of L-asparaginase isozymes [70]. L-asparaginase catalyzes the hydrolysis of L-asparagine, causing its rapid decline. L-asparagine is a non-essential amino acid that is synthetized in ordinary human cells by L-asparagine synthetase from aspartic acid. Neoplastic cells cannot synthesize L-asparagine due to the absence of this enzyme; hence, L-asparagine is an essential amino acid for these cells that must be obtained from external sources [71]. L-asparaginase is an enzyme currently used for acute lymphoblastic leukemia chemotherapy [72] and for the treatment of acute lymphoblastic leukemia [73], acute myeloid leukemia [74] and non-Hodgkin’s lymphoma [75]. L-asparaginase also has applications in the food industry and is used as a powerful attenuating agent to reduce acrylamide, which is carcinogenic [76]. Ebrahiminezhad et al. [71] found asparaginase activity in the green alga *Chlorella vulgaris*, while Lauritano et al. [77], for the first time, identified L-asparaginase in the dinoflagellate *Amphidinium carterae*. Asparaginase sequences for some microalgae are available in GenBank (https://www.ncbi.nlm.nih.gov/genbank/; accessed on 26 September 2022), including the diatoms *Phaeodactylum tricornutum*, *Fragilariopsis cylindrus* and *Thalassiosira pseudonana* and the eustigmatophyte *Microchloropsis gaditana*. L-asparaginase I was down-regulated in both *A. thurstonii* strains cultured in phosphate starvation compared with the control (log_2_FC of −4.21 with FDR < 3.09 × 10^−4^ for FE355 strain and log_2_FC of −0.88 with FDR < 1.84 × 10^−2^ for A4 strain), suggesting that the control condition is worthy of further bioactivity screening analysis and investigation.

Polyketide cyclase/dehydrase are a family of enzymes included in the star-related lipid-transfer (START)-like superfamily and involved in polyketide synthesis (https://www.ebi.ac.uk/interpro/entry/InterPro/IPR019587/#PUB00007207 accessed on 26 September 2022). Polyketides are a large group of natural biomolecules derived from repeated condensation of acetyl coenzyme A [11,78]. Polyketides are compounds with potential pharmaceutical applications due to their anti-cancer and anti-inflammatory properties [11,79,80,81]. The transcript of polyketide cyclase/dehydrase enzyme was down-regulated in both *A. thurstonii* strains cultivated in phosphate starvation conditions with respect to the control (log_2_FC −1.35 with FDR 1.03 × 10^−2^ for FE355 strain and log_2_FC −1.24 with FDR 1.80 × 10^−2^ for A4 strain). Bifunctional polyketide phosphatase/kinase, however, was down-regulated only in *A. thurstonii* A4 in phosphate starvation conditions (log_2_FC −2.12 with FDR 4.66 × 10^−3^).

1-deoxy-D-xylulose-5-phosphate synthase (fragment) has been reported to be correlated to the production of compounds with anti-cancer properties. In fact, it increases the accumulation of terpenoid indole alkaloid (TIA) in *Catharanthus roseus* hairy roots. In this plant, the TIA pathway produces two anti-cancer drugs, vinblastine and vincristine [82]. The transcript of 1-deoxy-D-xylulose-5-phosphate synthase (fragment) was down-regulated both in A4 (log_2_FC −1.36 with FDR 4.61 × 10^−3^) and FE355 (log_2_FC −1.65 with FDR 1.04 × 10^−2^) cultured in phosphate starvation.

Inositol polyphosphate 5-phosphatase INPP5B/F is an enzyme belonging to the family of inositol polyphosphate-5-phosphatases that may act on phosphate inositol or/and soluble phosphatides (IP) (https://www.genecards.org/cgi-bin/carddisp.pl?gene=INPP5B accessed on 22 October 2022). Inositol polyphosphate 5-phosphatase INPP5B/F is a key regulator of actin and B cell receptors (BCR) that are responsible for clustering and downstream signaling in antigen-stimulated B cell remodeling. B cells are the basis of the adaptative immune response and the appropriate activation of BCR is responsible for correct B cell development [83]. A critical mediator of this event is the actin cytoskeleton that undergoes reorganization upon receptor activation [84,85]. We found that INPP5B/F was expressed in *A. thurstonii* FE355 and was down-regulated in phosphate starvation conditions (log_2_FC −3.47 with FDR 4.19 × 10^−3^). On the contrary, INPP5B/F was not expressed in *A. thurstonii* A4 both in the control and the phosphate starvation conditions.

Catechol O-Methyltransferase (COMT) is an enzyme involved in the degradation pathway of catecholamines. According to the pathway commons database (https://apps.pathwaycommons.org/interactions/?source=COMT; accessed on 31 November 2022), COMT can have interactions with different proteins and transcription factors. For example, COMT is known to promote the expression of vitamin D3 receptor (VDR) (also called Nuclear Receptor Superfamily 1 group I member 1) (https://apps.pathwaycommons.org/interactions/?source=COMT accessed on 26 September 2022), whose downstream targets are involved in the metabolism of immune response and cancer. COMT also interacts with the pro-inflammatory cytokine tumor necrosis factor and with the tumor growth factor β1 (TGFβ1) that can modulate expression/activation of other growth factors, such as interferon gamma and tumor necrosis factor alpha, and is frequently up-regulated in tumor cells.

Digalactosyldiacylglycerol synthase is an enzyme involved in the synthesis of digalactosyldiacylglycerols (DGDGs). DGDGs have been reported to have anti-cancer functions by inhibiting DNA polymerase, suppressing cancer cell proliferation and having anti-angiogenesis properties. In addition, DGDGs have also been shown to have anti-inflammatory activity and to control appetite [86]. Digalactosyldiacylglycerol synthase was expressed in *A. thurstonii* A4 and in *A. thurstonii* FE355 in control conditions but was not expressed for both strains in phosphate starvation conditions.

1,2-diacylglycerol-3-beta-galactosyltransferase (monogalactosyldiacylglycerol synthase or MGDG) is an enzyme involved in glycerolipid metabolism [87]. Glycerolipids are a constituent of photosynthetic membranes, but they are also known for their anti-cancer activity [88,89]. This enzyme was expressed in *A. thurstonii* A4 and was down-regulated when cultured in phosphate starvation conditions (log_2_FC −2.75 with FDR 1.64 × 10^−3^). On the contrary, it was not found in the FE355 strain.

O-methyltransferase family 3, as discussed in Section 2.3.1, catalyzes the O-methylation of various secondary metabolites that have a broad spectrum of biological activities, including anti-inflammatory, anti-carcinogenic and anti-atherogenic properties [43]. Glycerolphospodieter phosphodiesterase, as already reported in Section 2.3.2, is involved in the synthesis of phospholipids known to have high anti-cancer activity [48].

## 3. Discussion

**Figure 2:**
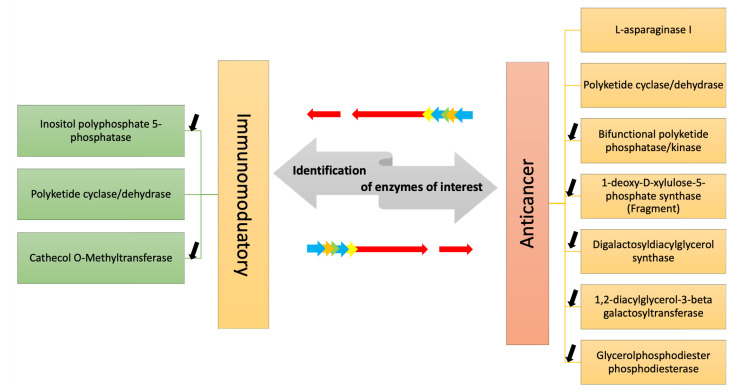
The figure shows the enzymes identified by transcriptome mining of two *A. thurstonii* strains, A4 and FE355, which may be involved in the synthesis/degradation of compounds with immunomodulatory and anti-cancer properties. Differential expression analysis of the transcripts in phosphate starvation for A4 and FE355 versus the control condition allowed to identify in which condition they were up-/down-regulated; black arrows indicate down-regulation, while no arrow indicates the presence of the transcript only in the control condition.

Considering the need for new drugs in order to expand the resources against cancer and other human pathologies, such as emerging viral and antibiotic-resistant infections, studies on metabolic pathways responsible for the synthesis of high-value compounds or enzymes with direct applications are increasing for possible genetic engineering approaches to meet the increasing industrial demand for new drugs. The current transcriptomic study allowed to identify in a marine diatom a series of transcripts coding enzymes involved in the synthesis/degradation/metabolism of compounds with immunomodulatory or anti-cancer activity, as well as an enzyme which is currently in use for cancer treatment (Figure 2). Overall, this study will help to increase knowledge about diatom transcript annotations and differential expression of key genes in different culturing conditions and will also give new insights on enzymatic pathways potentially involved in the generation of bioactive molecules.

Our study has identified a transcript encoding for L-asparaginase in the transcriptome of both *A. thurstonii* strains. Considering that this enzyme is currently in use for the treatment of leukemia, this promising result stimulates further experimentation of bioactivity screening on human leukemia cells for possible pharmaceutical applications of this diatom. Similarly, our analyses showed the presence of transcript coding for enzymes involved in the synthesis of polyketides, related to polyketide synthase (PKS). Type I PKS are large multifunctional proteins, with various domains, such as acyltransferase, β-ketosynthase, acyl carrier protein, β-ketoacyl reductase, enoyl reductase, methyl transferases, thioesterases and dehydrogenase domains [90]. PKS have been shown to be involved in the synthesis of toxins and other compounds with interesting ecological and biotechnological functions (e.g., anti-predator, allelopathic, anti-cancer anti-fungal activity and/or beneficial effects for the treatment of Alzheimer’s disease). The PKS-related enzymes found in the current study were down-regulated upon stress starvation exposure, as found also by Lauritano et al. for the dinoflagellate *Amphidinium carterae* [77], suggesting that the control condition is worthy of further analysis. Regarding the other enzymes, digalactosyldiacylglycerol synthase,1,2-diacylglycerol-3-beta-galactosyltransferase and glycerolphospodieter phosphodiesterase are noteworthy for their involvement in the synthesis of a glycerol-based lipid with recognized anti-cancer activity. In particular, digalactosyldiacylglycerol synthase is involved in the synthesis of digalactosyldiacylglycerols (DGDGs), compounds that act by inhibiting DNA polymerase and consequentially suppressing cancer cell proliferation and angiogenesis.

We also focused our attention on the identification of transcripts coding for enzymes involved in the synthesis of compounds with possible immunomodulatory activity: inositol polyphosphate 5-phosphatase (INPP5B/F), polyketide cyclase/dehydrase (described above) and catechol O-Methyltransferase (COMT). INPP5B/F is a key regulator of actin and B cell receptors (BCR) that are responsible for clustering and downstream signaling in antigen-stimulated B cells remodeling. Recently, INPP5B/F was reported to be significantly up-regulated in the microalgae *Phaeodactylum tricornutum* under low nitrogen culturing conditions, indicating that the phosphatidylinositol signaling system might play an important role in nitrogen-limited response as well [91]. Finally, catechol O-Methyltransferase (COMT) is involved in the degradation pathway of catecholamines by methylating them; it is also reported that it can have interactions with different proteins and transcription factors with immunomodulatory effects, as for example, the up-regulation of vitamin D3 receptor (VDR), whose downstream targets are involved in the metabolism of the immune response. Moreover, another immunomodulatory effect is the ability to interact with the pro-inflammatory cytokine tumor necrosis factor and with the tumor growth factor β1 (TGFβ1) by modulating the action of other growth factors.

*Asterionellopsis glacialis* has been considered for a long time as a cosmopolitan and eurytopic species, but Kaczmarska et al. [21] demonstrated that there was cryptic diversity and they identified four new species belonging to this group. In the current study, we used two *Asterionellopsis* strains coming from two different locations and, due to genotyping analyses, we found that both of these are *A. thurstonii*. Not much is known regarding the bioactivity of *Asterionellopsis* sp. and this study opens new possible bio-prospectives on this understudied genus.

We have previously shown that bioactivity may change not only depending on the studied species but also on the different strains of the same species and culturing conditions [17,32] in line with the findings of Ribalet et al. and Di Dato et al. [14,92]. Regarding different strain bioactivity, recently, Lauritano and co-workers showed that two strains of the marine diatom *Skeletonema marinoi* showed completely different activities as well as behaved differently in different culturing conditions [17]. In particular, the strain *S. marinoi* FE60 showed anti-cancer properties (against melanoma A2058 cells) and anti-bacterial properties only when cultivated in nitrogen starvation conditions, while the strain *S. marinoi* FE6 did not show anti-cancer properties but only anti-bacterial activity, only when cultivated in phosphate starvation conditions [26]. Similarly, Di Dato et al. [92] found differential expression in the enzymes involved in prostaglandin synthesis in the same two strains of the diatom *S. marinoi*. Prostaglandins are hormone-like compounds known to have a pivotal role in inflammatory responses.

## 4. Materials and Methods

### 4.1. Cell Culturing and Harvesting

The diatom *Asterionellopsis glacialis* s.l. FE355 was isolated in the Gulf of Naples, Italy, in 2014 and maintained at the Stazione Zoologica Anton Dohrn. *Asterionellopsis glacialis* s.l. A4 was sampled from the English Channel (waters off Dunkirk, Dunkirk, France) in 2017. Both strains were cultured in Guillard’s F/2 medium [93]. Experimental culturing was performed in triplicate for both replete (complete medium) and phosphate starvation conditions for each strain. For phosphate starvation, a low concentration of phosphate was used (0.5 μM PO_4_^2−^). Culturing was performed in 2-litre polycarbonate bottles, with constant bubbling with air filtered through 0.2 μm membrane filters in a climate chamber at 19 °C on a 12:12 h light:dark cycle at 100 μmol photons m^−2^ s^−1^. Initial cell concentration was 5000 cells/mL in each bottle. Cells were counted daily by fixing 2 mL of culture with Lugol (final concentration of about 2%, *v/v*) and counting cell numbers in a Bürker counting chamber under an Axioskop 2 microscope (20×) (Carl Zeiss GmbH, Oberkochen, Germany). Growth curves in control and phosphate starvation conditions are reported in Supplementary Material Figure S2. Culture aliquots (50 mL) were sampled in triplicate in the stationary growth phase (Day 5 for the control condition and Day 4 for the phosphate starvation condition), which has been previously reported as the condition where diatoms may produce bioactive secondary metabolites [14]. Aliquots were centrifuged for 15 min at 4 °C at 1900× *g* (Eppendorf, 5810R, Hamburg, Germany) and the corresponding pellets were then re-suspended in 500 µL of TRIZOL© (Invitrogen, Carlsbad, CA, USA), incubated for 2–3 min at 60 °C until completely dissolved and stored at −80 °C until RNA extraction. Another aliquot of 50 mL was also collected for each strain and centrifuged with the same conditions and the pellet stored directly at −80 °C for DNA extraction.

### 4.2. DNA Extraction and Genetic Characterization of the Strains

DNA extraction was performed as in Ruocco et al. [28]. Briefly, a lysis buffer containing cetyltrimethylammonium bromide (CTAB) 2% and 2-mercaptoethanol (2-ME, Sigma-Aldrich, Saint Louis, MO, USA) was used to disrupt cell membranes. RNAse was added (final concentration 200 μg/mL) and digestion was performed at 65 °C for 45 min. The extraction continued with two steps with chloroform/isoamyl alcohol (24:1), followed by a step with 1 volume of ice-cold isopropanol (100%) and glycogen for DNA precipitation at −20 °C overnight. DNA was then washed with 75% ethanol, centrifuged for 15 min, air-dried and suspended in 20 μL sterile water. Quantity and purity were estimated by using a NanoDrop spectrophotometer (ND-1000 UV–vis Spectrophotometer; NanoDrop Technologies, Wilmington, DE, USA). DNA integrity was evaluated by agarose gel electrophoresis. Based on the study published by Kaczmarska et al. [21], we amplified and sequenced the ITS region to discriminate our strains among the different species within the *A. glacialis* species complex. Polymerase chain reactions (PCRs) were carried out into a final volume of 20 µL, each containing 7–10 ng DNA, 2X Phire™ Plant Direct PCR Master Mix (Thermo Fisher Scientific, Waltham, MA, USA), 250 nM forward and reverse primers [94] and water to volume. PCR protocol was set to the following conditions: initial amplification at 98 °C for 30 s; 35 cycles at 98 °C for 5 s, 62 °C for 20 s and 72 °C for 30 s; final extension at 72 °C for 2 min. PCRs were visualized by electrophoresis in 1.5% agarose gel stained with SafeView™ Classic (ABM^®^, Richmond, BC, Canada) running in 0.5× TBE buffer. The amplified products were purified using 20% PEG (Polyethylene Glycol) 8000 (AppliChem, Darmstadt, Germany) and quantified on a 1.5% agarose gel. Sanger sequencing reactions were carried out using the BigDye Terminator Cycle sequencing kit (Applied Biosystems, Thermo Fisher Scientific, Waltham, MA, USA) and run on the Automated Capillary Electrophoresis Sequencer 3730 DNA Analyzer (Applied Biosystems, Thermo Fisher Scientific, Waltham, MA, USA) at the Molecular Biology and Sequencing facility at the Stazione Zoologica Anton Dohrn of Naples. The resulting sequences were aligned together with other ITS sequences of *A. glacialis* s.l. included in the study by Kaczmarska et al. [21], using the ClustalW algorithm [95] implemented in the software BioEdit [96]. The final alignment (Data S1) was manually trimmed at the 5′ and 3′ extremities to reduce the length of external gaps. The best fitting evolution model for ITS region was computed in jModelTest v2.1.3 [97] using the corrected Akaike information criterion [98]. A Bayesian phylogenetic tree was then inferred in MrBayes v3.2.6 [99] under the substitution model derived from the jModelTest analysis and running two replica runs and four chains for 1,000,000 generations and sampling chains every 1000 steps. Convergence and effective sample sizes (ESS) for all parameters (>200) were investigated in Tracer v.1.7 [100] and the first 10% of generations was discarded as burn-in. The majority-rule consensus tree was visualized in FigTree v1.4.3 (http://tree.bio.ed.ac.uk/software/figtree/; accessed on 24 November 2022).

### 4.3. RNA Extraction

RNA extraction was performed following the TRIZOL^®^ (Thermo Fisher Scientific, Waltham, MA, USA) manufacturer’s instructions. RNA quantity and quality were measured by using Nano-Drop (ND-1000 UV–Vis spectrophotometer; NanoDrop Technologies, Wilmington, DE, USA), monitoring the absorbance at 260 nm and the 260/280 nm and 260/230 nm ratios, respectively, as in Riccio et al. [9]. RNA samples were considered good when both ratios were approximately 2. RNA quality was also assessed on 1% agarose gel and by measuring the RNA integrity number (RIN) with Agilent 2100 Bioanalyzer (Agilent Technologies, Inc., Santa Clara, CA, USA). RNA with RIN > 8 was considered to be of high quality and further processed.

### 4.4. Sequencing, Assembly, Annotation and Differential Expression Analysis

Next generation sequencing experiments were performed by Genomix4life S.R.L. (Baronissi, SA, Italy). RNA concentration in each sample was assayed with a Nanodrop One (Thermo Fisher, Waltham, MA, USA) and its quality assessed with the TapeStation 4200 (Agilent Technologies, Santa Clara, CA, USA). Indexed libraries were prepared from 1 ug/ea purified RNA with Truseq Stranded mRNA according to the manufacturer’s instructions. Libraries were quantified using the TapeStation 4200 (Agilent Technologies, Inc., Santa Clara, CA, USA) and Qubit fluorometer (Invitrogen Co., Waltham, MA, USA), then pooled such that each index-tagged sample was present in equimolar amounts, with a final concentration of the pooled samples of 2 nM. The pooled samples were subject to cluster generation and sequencing using an Illumina NextSeq 550dx (Illumina) in a 2 × 75 paired-end format. The raw sequence files generated (.fastq files) underwent quality control analysis using FastQC (http://www.bioinformatics.babraham.ac.uk/projects/fastqc; accessed on 24 April 2020). The assembly was performed with Trinity (v2.8.6) (https://github.com/trinityrnaseq/trinityrnaseq/wiki; accessed on 24 April 2020) using the trimmed pair reads as input. Redundancy removal was performed with CD-HIT-EST with default parameters [37]. Kallisto (https://pachterlab.github.io/kallisto/about; accessed on 24 April 2020) [38] is a program used for quantifying abundances of transcripts from RNA-Seq data and, to remove all possible contamination, a BLASTN was performed. Transrate was used to extract the quality statistics about the assembly [39]. TransDecoder (https://github.com/TransDecoder/TransDecoder/wiki; accessed on 24 April 2020) was used to identify coding regions within transcript sequences and ORFs with homology to known proteins and PANNZER was used to perform the annotation of these proteins. The tool is designed to predict the functional description (DE) and GO classes. The NOISeq package is a non-parametric approach for the identification of differentially expressed genes from count data or previously normalized count data and was used for differential expression analysis. Gene ontology enrichment analysis (GOEA) was performed using Genomix4life S.R.L. in-house scripts based on the method described in Tian et al. [101] and setting a minimum FDR threshold of 0.05.

## 5. Conclusions

By using a transcriptomic approach, we annotated and identified for the first time in the diatom *A. thurstonii* transcript coding for enzymes involved in the synthesis/degradation/metabolism of compounds with potential immunomodulatory or anti-cancer activity. Interestingly, in the current study the identified enzymes of interest were almost all up-regulated in the control culturing condition, with respect to the phosphate starvation stressful condition. This suggests that anti-cancer compounds may be produced in greater quantities only in the control condition and not when the diatom cell is stressed. This can be confirmed by future chemical analyses to identify the compounds potentially produced by these enzymes and by screenings to check for possible bioactivities. The annotation of these transcripts in *A. thurstonii* may help to predict and annotate the same enzymes in other microalgae. Finally, the transcripts identified in this study can be seen as targets for possible heterologous expression production of bioactive molecules of interest in order to meet the increasing demand by industries for new drugs to treat cancer and other human diseases.

## Figures and Tables

**Table 1:** Number of transcripts across the filters.

	Trinity (Raw Assembly)	CD-HIT	KALLISTO	BLAST
FE355	44,430	42,571	42,527	40,493
A4	46,835	44,637	44,586	41,698

**Table 2:** Assembly statistics for *Asterionellopsis thurstonii* strain FE355 and A4, respectively.

Parameters	FE355 Assembly	A4 Assembly
Total transcripts	40,493	41,698
Percent GC	41.46	41.37
Contig N50	1090	1127
Median contig length	558	597
Average contig length	771.53	802.21
Total assembled bases	31,241,667	33,450,597

**Table 3:** Number of differentially expressed transcripts. Conditions in bold are considered as reference transcriptome for each comparison.

Comparison	Total DE Transcripts	Up-Regulated	Down-Regulated
A4_ phosphate starvation_vs._**A4_Control**	3669	2276	1393
FE355_ phosphate starvation_vs._**FE355_Control**	3358	2206	1152
A4_Control_vs._**FE355_Control**	1628	441	1187

**Table 4:** First ten up-regulated genes and first ten down-regulated genes in *Asterionellopsis thurstonii* A4 in P-starvation.

Up-Regulated Genes	log_2_FC	Down-Regulated Genes	log_2_FC
O-methyltransferase family 3	+8.41	Peptidyl-prolyl cis–trans isomerase	−6.96
Alkaline phosphatase	+7.99	Phosphoglycerate mutase-like protein	−6.40
Catechol O-methyltransferase domain-containing protein 1	+7.91	Pentapeptide repeat-containing protein	−6.24
Glycerophosphodiester phosphodiesterase	+7.62	Thylakoid lumenal 17.4 kDa protein, chloroplastic	−6.15
Nucleotide-sugar transporter-domain-containing protein	+7.03	3-beta hydroxysteroid dehydrogenase/isomerase family	−5.54
Acyl-CoA thioesterase	+6.62	3-oxoacyl-ACP synthase (Fragment)	−5.59
Nucleotide-sugar transporter-domain-containing protein	+6.49	DUF1995 domain-containing protein (Fragment)	−5.46
Glycerophosphodiester phosphodiesterase (Fragment)	+6.44	Peptidyl-prolyl cis–trans isomerase, chloroplastic-like isoform X2	−5.39
Solute carrier family 35, member C2	+6.25	Calcium/calmodulin dependent protein kinase II association-domain protein	−5.29
DNA degradation protein EddB	+6.20	Ternary protein-Dna Complex1 (Fragment)	−5.17

**Table 5:** First ten up-regulated genes and the first ten down-regulated genes in *Asterionellopsis thurstonii* FE355 in P-starvation.

Up-Regulated	log_2_FC	Down-Regulated	log_2_FC
Catechol O-methyltransferase domain-containing protein 1	+8.50	Fucoxanthin chlorophyll a/c protein	−7.87
Glycerophosphodiester phosphodiesterase (Fragment)	+8.01	Light-harvesting complex protein LHCC4 (fragment)	−7.19
ATP-binding cassette	+7.96	Light-harvesting complex I polypeptide	−7.02
Glycerophosphodiester phosphodiesterase	+7.70	Protochlorophyllide oxidoreductase	−6.86
SPX domain-containing protein	+6.12	Chloroplast light harvesting protein isoform 7	−6.84
Transcriptional regulator MraZ	+5.99	Pyruvate carboxylase (fragment)	−6.54
Extracellular nuclease, putative	+5.89	Iron-only hydrogenase group A	−6.51
Translation initiation factor IF-3	+5.87	Light harvesting complex protein 10	−6.40
Ribosomal protein l12	+5.67	Chloroplast light harvesting protein isoform 5	−6.39
Cell division cycle 2, cofactor of APC complex	+5.61	Fucoxanthin-chlorophyll a/c light-harvesting protein (fragment)	−6.34

## Data Availability

Transcriptome sequences were deposited in the public database SRA (code PRJNA701376).

## Supplementary Materials

The following supporting information can be downloaded at: https://www.mdpi.com/article/10.3390/md21020126/s1, Figure S1: Bayesian consensus tree based on the analysis of ITS region (ITS1, 5.8S and ITS2); Figure S2: Growth curves in (a) control and (b) phosphate starvation conditions; Data S1: ITS alignment in nexus format; Table S1: Transcript annotation for FE355; Table S2: Transcript annotation for A4; Table S3: Differential expression of A4 in phosphate starvation versus its control condition; Table S4: Differential expression of FE355 in phosphate starvation versus its control condition; Table S5: Differential expression of A4 in control condition versus FE355 in control condition; Table S6: The table shows the first seven categories of Gene Ontology (GO) involved in biological process, molecular function and cellular component that were up-regulated; Table S7: The table shows the first seven categories of Gene Ontology (GO) involved in biological process, molecular function and cellular component that were down-regulated.
