# Supplementary material for: Transcriptome Sequencing of the Diatom Asterionellopsis thurstonii and In Silico Identification of Enzymes Potentially Involved in the Synthesis of Bioactive Molecules
Source: Mar Drugs. 2023 Feb 15;21(2):126. doi: 10.3390/md21020126 (PMC9959416; doi:10.3390/md21020126)

Figure S1. Bayesian consensus tree based on the analysis of ITS region (ITS1, 5.8S and ITS2). Number at nodes are posterior probabilities.

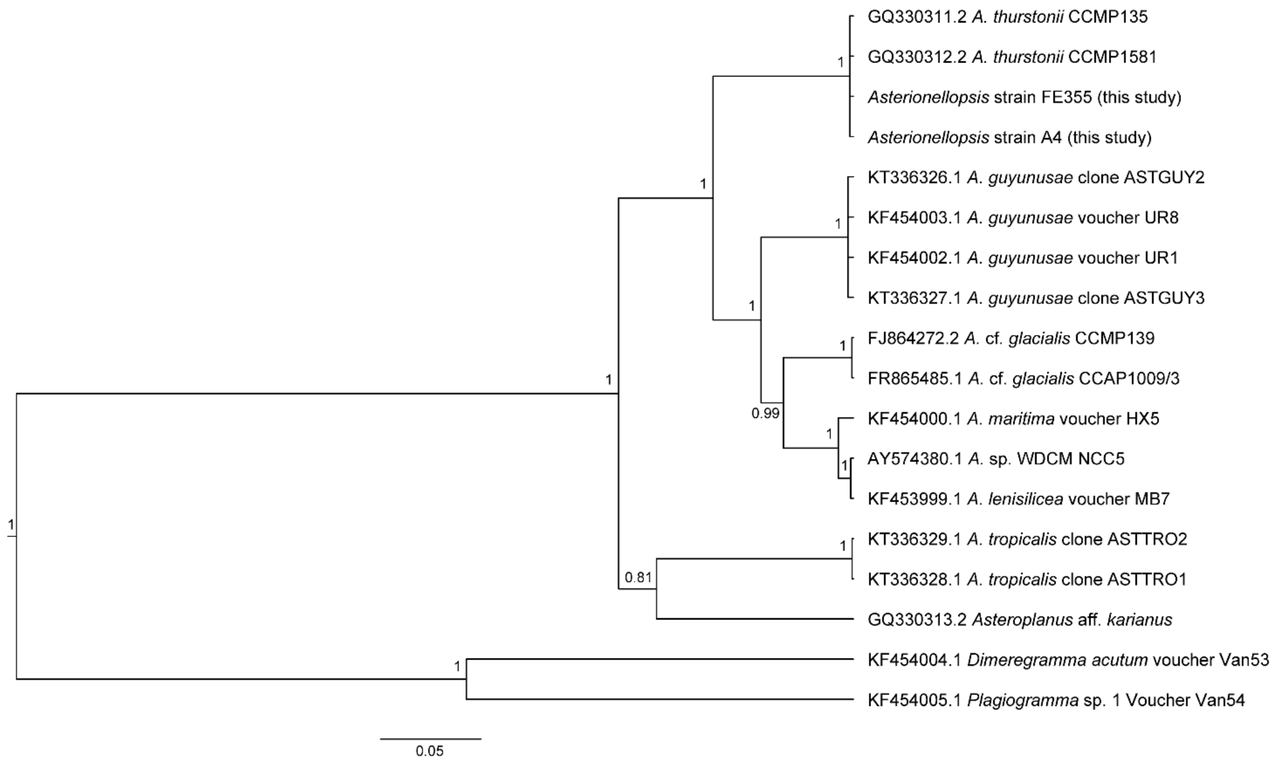

Supplement: Supplementary file 1 [file marinedrugs-21-00126-s001.zip › marinedrugs-2124680/Figure S1.pdf]
