# Supplementary material for: Transcriptome Sequencing of the Diatom Asterionellopsis thurstonii and In Silico Identification of Enzymes Potentially Involved in the Synthesis of Bioactive Molecules
Source: Mar Drugs. 2023 Feb 15;21(2):126. doi: 10.3390/md21020126 (PMC9959416; doi:10.3390/md21020126)
Supplement: Supplementary file 1 [file marinedrugs-21-00126-s001.zip › marinedrugs-2124680/Tables S6 and S7 corrette finali.pdf]

Table S6. The table shows the first seven categories of Gene Ontology (GO) involved in biological process, molecular function and cellular component that were up-regulated.

**GOEA up-regulated A4_Control_vs_FE355_Control**			
**GO Class**	**GO Description**	**FDR**	**Enrichment score**
Biological Process	Fatty acid biosynthetic process	2.37E-03	6.81
Biological Process	Signal transduction	3.86E-03	4.63
Biological Process	Phosphorylation	3.87E-02	2.17
Biological Process	Transmembrane transport	1.57E-01	1.45
Biological Process	Oxidation-reduction process	2.10E-01	1.21
Biological Process	Proteolysis	2.38E-01	1.25
Biological Process	Protein phosphorylation	2.68E-01	1.20
Cellular component	Bounding membrane of organelle	1.18E-03	9.84
Cellular component	Mitochondrial matrix	4.97E-03	5.37
Cellular component	Intracellular membrane-bounded organelle	5.76E-03	3.56
Cellular component	Endoplasmic reticulum	5.57E-02	1.97
Cellular component	Integral component of membrane	1.07E-01	1.14
Cellular component	Cell	3.19E-01	1.10
Cellular component	Cytoplasm	4.13E-01	1.02
Molecular function	Lipid binding	1.18E-03	9.84
Molecular function	Oxidoreductase activity	7.30E-03	2.44
Molecular function	Transferase activity	2.54E-02	2.68
Molecular function	Kinase activity	2.59E-02	2.65
Molecular function	ATP binding	5.88E-02	1.41
Molecular function	Metal ion binding	7.49E-02	1.51
Molecular function	Hydrolase activity	2.19E-01	1.32
**GOAE up-regulated A4_phosphate starvation_vs_A4_Control**			
**GO Class**	**GO Description**	**FDR**	**Enrichment score**
Biological process	Glucose metabolic process	2.94E-03	7.18
Biological process	Transmembrane receptor protein serine/threonine kinase signaling pathway	4.60E-03	6.39
Biological process	Regulation of cyclin-dependent protein serine/threonine kinase activity	6.21E-03	4.79
Biological process	Negative regulation of apoptotic process	3.38E-03	4.56
Biological process	Signal transduction	1.12E-02	2.74
Biological process	Regulation of transcription from RNA polymerase II promoter	1.73E-02	2.74
Biological process	Protein phosphorylation	8.86E-03	1.76
Cellular component	Host cell nucleus	5.08E-05	3.72
Cellular component	Extracellular region	3.93E-04	3.28
Cellular component	Chloroplast thylakoid membrane	1.53E-01	1.53
Cellular component	Nucleus	8.31E-02	1.29

Cellular component	Intracellular membrane-bounded organelle	3.27E-01	1.13
Cellular component	Cytoplasm	2.70E-01	1.11
Cellular component	Nucleoplasm	4.59E-01	0.91
Molecular function	Glucose-6-phosphate dehydrogenase activity	2.69E-04	11.50
Molecular function	Glycerophosphodiester phosphodiesterase activity	5.62E-05	10.95
Molecular function	Cyclic-di-GMP binding	3.02E-04	8.52
Molecular function	Phospholipase A2 activity	1.62E-03	8.21
Molecular function	S-adenosylmethionine-dependent methyltransferase activity	2.37E-03	5.90
Molecular function	Chitin binding	1.94E-03	4.42
Molecular function	Sequence-specific DNA binding	1.94E-03	3.94

GOAE up-regulated FE355_phosphate starvation_vs_FE355_Control

GO Class	Go Description	FDR	Enrichment score
Biological process	Cysteine transport	1.16E-04	11.00
Biologica process	Protein repair	5.16E-04	8.80
Biological process	Phospholipid scrambling	3.58E-04	7.34
Biological process	Ribosome assembly	2.94E-03	6.29
Biological process	rRNA metabolic process	3.49E-03	4.89
Biological process	Ribosomal large subunit biogenesis	3.49E-03	4.89
Biological process	Translation	1.66E-04	2.58
Cellular component	Glycosome	5.16E-04	8.80
Cellular component	Pre-ribosome, large subunit precursor	5.66E-05	5.71
Cellular component	Proton-transporting V-type ATPase, V0 domain	8.67E-03	4.89
Cellular component	Small ribosomal subunit	1.23E-02	4.40
Cellular component	Intracellular non-membrane-bounded organelle	3.69E-03	3.67
Cellular component	Nucleolus	2.86E-04	2.82
Cellular component	Ribosome	2.98E-03	2.24
Molecular function	Phosphatidylserine binding	1.16E-04	11.00
Molecular function	2-alkenal reductase [NAD(P)] activity	5.16E-04	8.80
Molecular function	Enhancer binding	5.16E-04	8.80
Molecular function	RNA polymerase II regulatory region DNA binding	5.16E-04	8.80
Molecular function	Calcium-dependent phospholipid binding	9.35E-07	8.56
Molecular function	Phospholipid scramblase activity	3.58E-04	7.34
Molecular function	Structural constituent of ribosome	2.35E-05	2.69

Table S7. The table shows the first seven categories of Gene Ontology (GO) involved in biological process, molecular function and cellular component that were down-regulated.

**GOEA down-regulated A4_Control_vs_FE355_Control**			
**GO Class**	**GO Description**	**FDR**	**Enrichment score**
Biological process	Toll signaling pathway	3.19E-05	14.42
Biological process	Negative regulation of cell growth	3.19E-05	14.42
Biological process	Ribosomal small subunit assembly	1.50E-04	11.54
Biological process	Positive regulation of oxidative phosphorylation	1.50E-04	11.54
Biological process	Cell wall organization	3.23E-04	7.69
Biological process	Proteolysis involved in cellular protein catabolic process	1.05E-04	7.39
Biological process	Translation	3.16E-09	4.09
Cellular component	Synapse	4.25E-04	9.61
Cellular component	Proteasome core complex, alpha-subunit complex	8.92E-04	8.24
Cellular component	Cytosolic ribosome	8.92E-04	8.24
Cellular component	Proteasome complex	2.74E-04	6.41
Cellular component	Proteasome core complex	8.76E-04	6.41
Cellular component	Ribonucleoprotein complex	7.36E-08	5.38
Cellular component	Ribosome	2.77E-07	3.66
Molecular function	Proteasome binding	3.19E-05	14.42
Molecular function	Lactoylglutathione lyase activity	1.50E-04	11.54
Molecular function	Phosphoglycerate kinase activity	4.25E-04	9.61
Molecular function	RNA polymerase II transcription factor activity, sequence-specific DNA binding	8.92E-04	8.24
Molecular function	Threonine-type peptidase activity	6.04E-06	7.48
Molecular function	Endopeptidase activity	6.99E-04	4.21
Molecular function	Structural constituent of ribosome	5.83E-09	3.85
**GOAE down-regulated A4_phosphate starvation_vs_A4_Control**			
**GO Class**	**GO Description**	**FDR**	**Enrichment score**
Biological process	Response to high light intensity	1.16E-05	10.71
Biological process	Photosystem II stabilization	1.52E-04	10.04
Biological process	Carbohydrate derivative biosynthetic process	1.52E-04	10.04

Biological process	NAD biosynthetic process	2.08E-05	8.37
Biological process	Response to light stimulus	6.79E-10	7.25
Biological process	Response to cold	5.06E-04	6.69
Biological process	Oxidation-reduction process	3.56E-04	1.50
Cellular component	Photosystem II oxygen evolving complex	5.06E-04	6.69
Cellular component	Late endosome	1.79E-03	6.69
Cellular component	Photosystem II	6.06E-05	5.51
Cellular component	Photosystem I	2.31E-04	5.35
Cellular component	Plastid stroma	3.07E-03	4.87
Cellular component	Thylakoid membrane	4.85E-03	4.46
Cellular component	Chloroplast	3.06E-04	2.88
Molecular function	Protein transmembrane transporter activity	3.07E-05	10.04
Molecular function	Glutaminase activity	1.44E-04	8.03
Molecular function	Pyruvate kinase activity	4.08E-04	6.69
Molecular function	Structural constituent of cuticle	4.08E-04	6.69
Molecular function	Potassium ion binding	1.68E-03	5.02
Molecular function	Ubiquitin binding	7.02E-04	4.18
Molecular function	2 iron, 2 sulfur cluster binding	2.50E-06	4.12

GOAE down-regulated FE355_phosphate starvation_vs_FE355_Control

**GO Class**	**GO Description**	**FDR**	**Enrichment score**
Biological process	Cell morphogenesis	6.38E-04	8.05
Biological process	Glyoxylate cycle	6.38E-04	8.05
Biological process	Response to light stimulus	5.83E-12	6.91
Biological process	Protein refolding	1.73E-03	6.71
Biological process	Proteasome-mediated ubiquitin-dependent protein catabolic process	6.45E-05	6.19
Biological process	tRNA aminoacylation for protein translation	9.73E-04	5.96
Biological process	Tricarboxylic acid cycle	4.10E-04	3.98
Cellular component	Phagocytic vesicle	6.38E-04	8.05
Cellular component	Photosystem II	5.59E-07	6.71
Cellular component	Axon	1.73E-03	6.71
Cellular component	Photosystem I	3.09E-05	5.87
Cellular component	Ribonucleoprotein granule	3.68E-03	5.75
Cellular component	Cytosol	1.73E-03	1.68
Cellular component	Cytoplasm	6.64E-04	1.55
Molecular function	aminoacyl-tRNA editing activity	1.04E-05	10.73

Molecular function	Oxoglutarate dehydrogenase (succinyl-transferring) activity	6.38E-04	8.05
Molecular function	Inorganic diphosphatase activity	1.89E-04	7.67
Molecular function	Hydrogen-translocating pyrophosphatase activity	1.89E-04	7.67
Molecular function	Single-stranded DNA binding	3.68E-03	5.75
Molecular function	ATP binding	1.89E-04	1.48
Molecular function	Metal ion binding	5.79E-04	1.62
