# Supplementary figures and images for: Transcriptome Sequencing of the Diatom Asterionellopsis thurstonii and In Silico Identification of Enzymes Potentially Involved in the Synthesis of Bioactive Molecules
Source: Mar Drugs. 2023 Feb 15;21(2):126. doi: 10.3390/md21020126 (PMC9959416; doi:10.3390/md21020126)

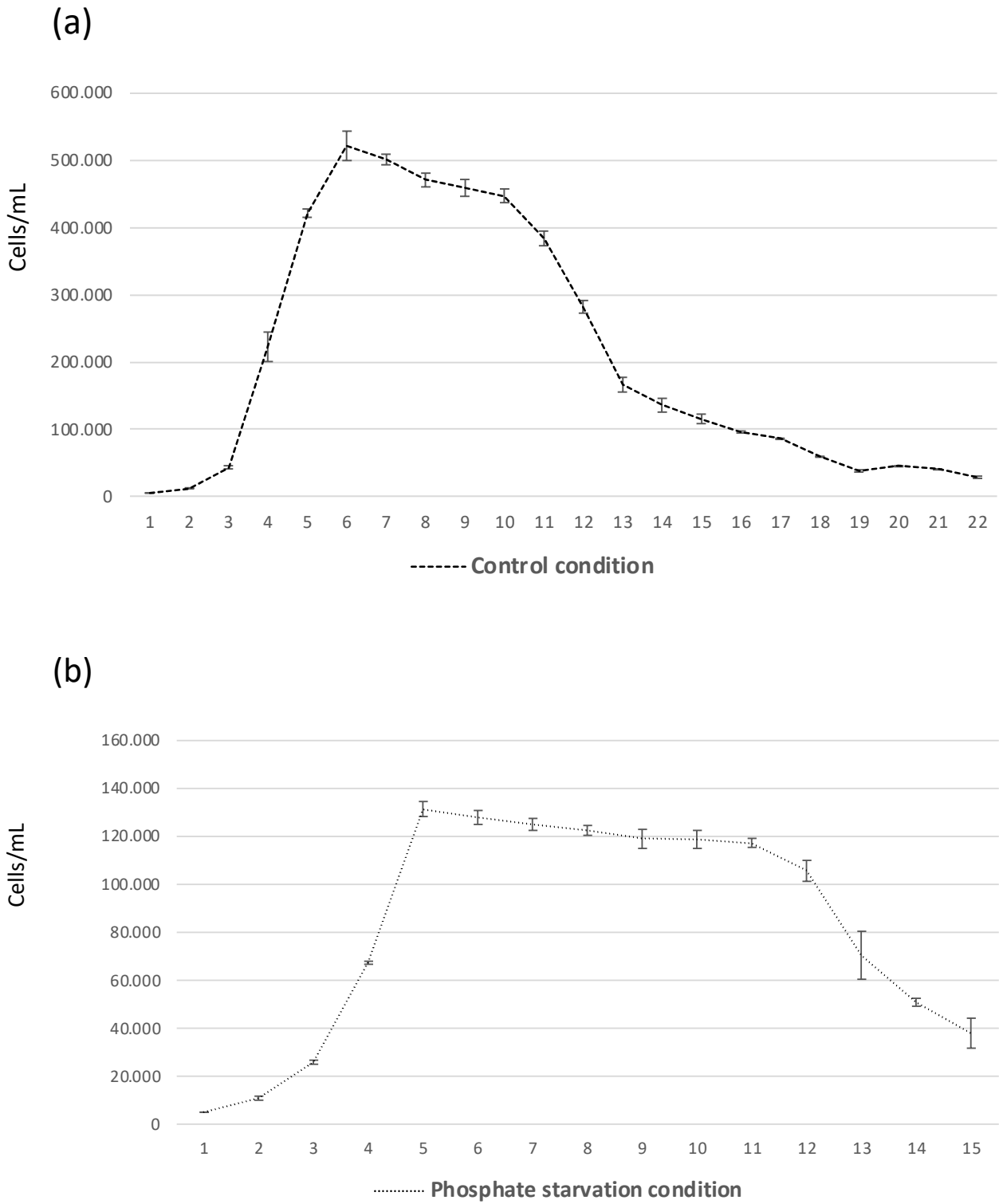

**Figure S2.** Growth curves in (a) control and (b) phosphate starvation conditions

Supplement: Supplementary file 1 [file marinedrugs-21-00126-s001.zip › marinedrugs-2124680/Figure S2.pdf]
